# Potential tumor biomarkers identified in ovarian cyst fluid by quantitative proteomic analysis, iTRAQ

**DOI:** 10.1186/1559-0275-10-4

**Published:** 2013-04-04

**Authors:** Björg Kristjansdottir, Kristina Levan, Karolina Partheen, Elisabet Carlsohn, Karin Sundfeldt

**Affiliations:** 1 Institute of Clinical Sciences, Department of Obstetrics and Gynecology, University of Gothenburg, Gothenburg S-413 45, Sweden; 2 Proteomics Core Facility at Sahlgrenska Academy, Gothenburg University, Gothenburg, Sweden

**Keywords:** Ovarian adenocarcinoma, Ovarian cyst fluid, Tumor biomarker, Mass spectrometry, iTRAQ

## Abstract

**Background:**

Epithelial-derived ovarian adenocarcinoma (EOC) is the most deadly gynecologic tumor, and the principle cause of the poor survival rate is diagnosis at a late stage. Screening and diagnostic biomarkers with acceptable specificity and sensitivity are lacking. Ovarian cyst fluid should harbor early ovarian cancer biomarkers because of its closeness to the tumor. We investigated ovarian cyst fluid as a source for discovering biomarkers for use in the diagnosis of EOC.

**Results:**

Using quantitative mass spectrometry, iTRAQ MS, we identified 837 proteins in cyst fluid from benign, EOC stage I, and EOC stage III. Only patients of serous histology were included in the study. Comparing the benign (n = 5) with the malignant (n = 10) group, 87 of the proteins were significantly (p < 0.05) differentially expressed. Two proteins, serum amyloid A-4 (SAA4) and astacin-like metalloendopeptidase (ASTL), were selected for verification of the iTRAQ method and external validation with immunoblot in a larger cohort with mixed histology, in plasma (n = 68), and cyst fluid (n = 68). The protein selections were based on either high significance and high fold change or abundant appearance and several peptide recognitions in the sample sets (p = 0.04, FC = 1.95) and (p < 0.001, FC = 8.48) for SAA4 and ASTL respectively. Both were found to be significantly expressed (p < 0.05), but the methods did not correlate concerning ASTL.

**Conclusions:**

Fluid from ovarian cysts connected directly to the primary tumor harbor many possible new tumor-specific biomarkers. We have identified 87 differentially expressed proteins and validated two candidates to verify the iTRAQ method. However several of the proteins are of interest for validation in a larger setting.

## Background

Epithelial ovarian carcinoma (EOC) is the fifth most common cause of cancer deaths among women in Western Europe and the U.S., and unfortunately the majority of patients are diagnosed in late stages with a poor prognosis [1]. The five-year relative survival ranges from 90% for patients diagnosed with stage I tumors to only 35% for patients with advanced staged tumors, III or IV, according to the International Federation of Gynaecology and Obstetrics (FIGO) [2,3]. Thus, early detection seems to be the single most important factor for improving survival rates for patients with EOC.

Ovarian tumors commonly grow in cystic formations, and the majority of these cysts are benign and therefore harmless. Because no reliable diagnostic tests or imaging techniques are able to distinguish between a benign and a malignant cyst, approximately seven patients with benign lesions are operated for every ovarian cancer found [4]. Improving early diagnosis can help avoid unnecessary operations. Using CA-125 as a biomarker for early detection has been thoroughly investigated in several studies [5-8]. However, CA-125 is often falsely negative in fertile women with EOC and in early stage EOC and CA-125 is positive in a variety of benign diseases and therefore not sensitive enough to be used for general screening [9-12]. Among hundreds of suggested new biomarkers, human epididymis protein 4 (HE4) is a strong candidate for detection of EOC [13,14]. Reports indicate that HE4 and CA-125 in serum samples detect ovarian cancer equally, while HE4 has a better capacity to distinguish benign disease in fertile women from those with malignant tumors. Studies also indicate that HE4 is better at identifying early stage disease than CA-125 [14-16].

Proteomic profiling using mass spectrometry (MS) has been employed to detect biomarkers in serum and urine from patients with ovarian cancer [17]. Single biomarkers have previously been found in ovarian cyst fluid with different expression in benign versus malignant histology [18,19]. Mass-spectrometry-based quantitative proteomics has gained popularity in recent years because it enables both identifying proteins and studying changes in protein abundance in biological samples. Moreover, methods for quantitative MS–based proteomics using isobaric tags such as iTRAQ and TMT provide the advantages of enabling samples to be mixed into one reaction and several samples (up to seven) run together with a reference sample under identical conditions. These methods have been used in only a few EOC investigations. Boylan et al. performed an iTRAQ analysis in an attempt to identify biomarker candidates in ovarian cancer serum, and Gagné et al. have studied differences in protein expression between two EOC cell lines [20,21]. In addition, a study of tissue biopsies analysed with iTRAQ was recently published [22].

Epithelial-derived ovarian cysts are filled with fluid that is secreted from the local microenvironment, tumors cells and stroma. The ovarian cyst fluid contains proteins at much higher concentrations than in the blood [18,19]. Pathological changes within the ovaries should be reflected in the proteomic patterns of these cyst fluids, and the changes may differ between benign and malignant ovarian tumors of different grades and stages. Similar studies have been performed for improving the diagnosis of pancreatic cysts [23].

In an attempt to identify potential novel biomarkers that give the ability to distinguish malignant from benign cysts in patients diagnosed with a suspicious ovarian cystic pelvic mass, we analyzed a selection of immunodepleted cyst fluids from serous tumors with iTRAQ MS in an LTQ–Orbitrap XL mass spectrometer. We then investigated the identity of significant proteins and validated potentially useful biomarkers in a larger set of cyst fluids and serum samples with mixed histology.

## Results

### 32 proteins were differentially expressed in the iTRAQ MS analysis

**Figure 1:**
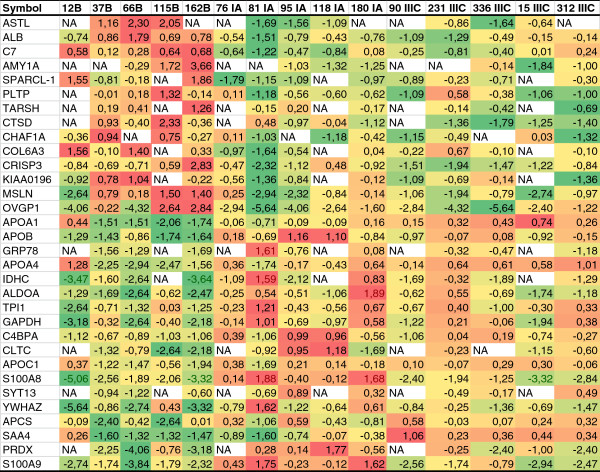
**Proteins detected with iTRAQ analysis in cyst fluid from serous ovarian cysts.** The 32 proteins that were considered to be differentially expressed in benign and malignant cysts are shown; relative protein levels are in logarithmic scale. B=benign, IA=stage IA, and IIIA=stage IIIC. The green color indicates lower and the red higher expression levels in relation to the other samples analyzed.

In total, 837 proteins were detected with iTRAQ MS analysis in the ovarian cyst fluids. Cyst fluids were run in five sets with three samples in each set (one benign, one EOC stage I, and one EOC stage III) (Table 1). Among them, we found 87 proteins that were significantly (p < 0.05) differentially expressed between the serous adenoma (benign) and serous adenocarcinoma (malignant) samples. Proteins identified by single or two peptides only, fold change <1.8, and all immunoglobulins (Ig) were excluded. The relative expression of the remaining 32 proteins in each cyst fluid sample is displayed in Figure 1. Proteins were divided into less expressed or more expressed in malignant samples compared to benign samples. Accession number, description, statistical evaluation, and fold change ranging from 1.80 to 8.48 are presented for each protein (Table 2). These proteins represent different functions in cell regulation and association with cancer or inflammatory response. Apart from significance and fold change, each protein was evaluated according to the number of appearances in the sample sets and peptide recognition hits. Of the 837 total proteins identified, 23% were identified in five sets, 29% were found in two-four sets, and 45% were uniquely expressed. Fold change > 2.0 were found in 75% of the proteins separating benign from malignant. Of these 32 proteins, 59% (n = 19) were expressed in five sets, 25% (n = 8) in four sets, and 16% (n = 5) in only three sets. Of these 32 proteins, 12% (n = 4) were recognized by 44–219 peptides in each set, and all four were identified as albumin or apolipoproteins, commonly detected in serum. The majority, 56% (n = 18), were recognized by 2–30 peptides in each set, while 32% (n = 10) were detected by only 1–3 peptides.

**Table 1:** Cyst fluid samples analyzed with iTRAQ

	**Benign (n = 5)**	**Stage I (n = 5)**	**Stage III (n = 5)**	**Malignant total (n = 10)**
Mean Age (year, (range))	71 (52–86)	60 (48–60)	65 (49–84)	63 (48–84)
Differentiation				
High	-	2	0	2
Moderate	-	1	0	1
Poor	-	2	5	7

Only serous adenoma and serous EOC were included, five benign, five stage IA, and five stage IIIC.

**Table 2:** Proteins detected with iTRAQ that are differentially expressed comparing benign and malignant serous ovarian cyst samples

**Gene symbol**	**Accession**	**Protein name**	**p-value**	**Fold change**
**Less expressed in malignant samples**				
ASTL	Q6HA08	Astacin-like metalloendopeptidase	< 0.001	8.48
ALB	P02768	Albumin	0.001	2.63
C7	P10643	Complement component 7	0.002	1.85
AMY1A	P04745	Amylase, alpha 1A	0.01	6.93
SPARCL1	Q14515	SPARC-like 1 (hevin)	0.01	2.82
PLTP	P55058	Phospholipid transfer protein	0.02	1.80
ABI3BP	Q7Z7G0	Target of Nesh-SH3 (TARSH)	0.02	1.80
CTSD	P07339	Cathepsin D	0.02	2.95
CHAF1A	Q13111	Chromatin assembly factor 1, subunit A	0.03	1.93
COL6A3	P12111	Collagen, type VI, alpha 3	0.03	2.22
CRISP3	P54108	Cysteine-rich secretory protein 3	0.04	2.59
KIAA0196	Q12768	Strumpellin (STRUM)	0.04	1.92
MSLN	Q13421	Mesothelin	0.04	3.02
OVGP1	Q12889	oviductal glycoprotein 1	0.05	6.53
**More expressed in malignant samples**				
APOA1	P02647	Apolipoprotein A-I	0.002	2.62
APOB	P04114	Apolipoprotein B	0.004	2.43
HSPA5 /GRP78	P11021	heat shock 70 kDa protein 5 (glucose-regulated protein, 78 kDa)	0.005	3.34
APOA4	P06727	Apolipoprotein A-IV	0.02	3.31
IDHC	O75874	Isocitrate dehydrogenase 1 (NADP+)	0.02	4.27
ALDOA	P04075	Aldolase A, fructose-bisphosphate	0.02	2.70
TPI1	P60174	Triosephosphate isomerase 1	0.02	2.18
GAPDH	P04406	Glyceraldehyde-3-phosphate dehydrogenase	0.03	2.76
C4BPA	P04003	Complement component 4 binding protein, alpha	0.03	1.80
CLTC	Q00610	Clathrin, heavy chain	0.03	2.69
APOC1	P02654	Apolipoprotein C-I	0.04	1.87
S100A8	P05109	S100 calcium binding protein A8	0.04	4.35
SYT13	Q7L8C5	Synaptotagmin XIII	0.04	1.97
YWHAZ	P63104	Tyrosine 3-monooxygenase/tryptophan 5-mono-oxygenase activation protein, zeta polypeptide	0.04	3.79
APCS	P02743	Amyloid P-component , serum	0.04	2.12
SAA4	P35542	Serum amyloid A-4, constitutive	0.04	1.95
PRDX2	P32119	Peroxiredoxin 2	0.04	4.03
S100A9	P06702	S100 calcium binding protein A9	0.05	3.43

**Figure 2:**
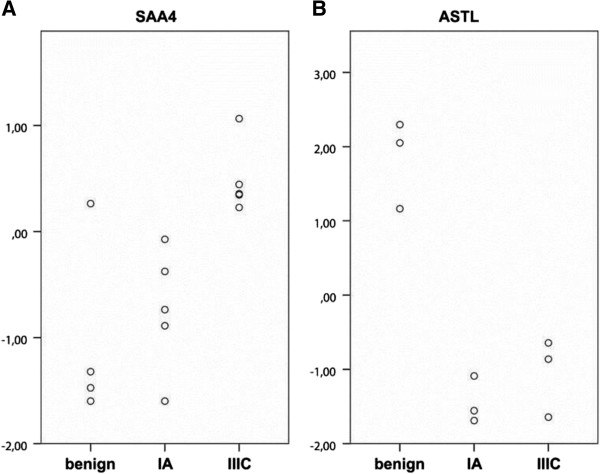
**iTRAQ cyst fluid analysis on SAA4 and ASTL; relative protein levels in benign, stage IA, and stage IIIC for A) SAA4 and B) ASTL in serous ovarian cyst fluid.** Both proteins showed a significant difference in expression levels between benign and malignant (stage IA and IIIC together) samples (ASTL p<0.001 and SAA4 p=0.04). When benign samples are compared to stage IA, there is still a significant difference in ASTL levels (p=0.001).

For verification of the iTRAQ method and external validation in a larger cohort, two proteins were selected for immunoblot validation. The protein selection was based on significance and high fold change between benign and malignant tumors or abundant appearance and several peptide recognitions in the sample sets (Figure 2, Table 2). Serum amyloid A-4 (SAA4) was increased in the malignant samples and detected in all five sets with 5–11 peptides in each set, but with lower stringency and fold change (p = 0.04, FC = 1.95) than astacin-like metalloendopeptidase (ASTL) (p < 0.001 and FC = 8.48). ASTL was decreased in the malignant cyst fluid compared to the benign, and was detected in three sets with 1–3 peptides in each set. The low number of identified peptides may indicate a more uncertain result for ASTL (Table 2).

In addition S100A8 (Calgranulin A) and S100A9 (Calgranulin B), proteins previously described in several tumor types, both displayed higher expression levels in the malignant samples compared to the benign samples (FC = 4.35 and 3.43 respectively) (Table 3). The iTRAQ analysis also identified SPARC-like protein 1 (SPARCL1), described as having the capacity to suppress tumors, since expression is higher in the benign samples (FC = 2.82), and serum amyloid P-component (FC = 2.12), recently found by iTRAQ in ovarian tumor serum and tissue biopsies [20,22].

**Table 3:** Sample characteristics of cyst fluid and plasma samples analyzed with immunoblot

	**Benign (n = 32)**	**Malignant (n = 36)**	**Stage I (n = 18)**	**Stage III (n = 17)**	**Stage IV (n = 1)**
Mean Age (year, (range))	57 (16–86)	59 (40–80)			
Simple	8 (25%)				
Endometrioma	6 (19%)				
Serous	12 (38%)	18 (50%)	7 (39%)	11 (65%)	
Mucinous	6 (19%)	6 (17%)	4 (22%)	1 (6%)	1 (100%)
Endometrioid		6 (17%)	3 (17%)	3 (18%)	
Clear cell		6 (17%)	4 (22%)	2 (12%)	

68 samples with mixed histology were included.

### External validation by immunoblot in 136 samples from 68 patients

To further study the results found in the iTRAQ analysis, we performed immunoblot analysis to establish the expression levels of a set of proteins in both ovarian cyst fluid and plasma (Table 3). As discussed in the paragraph above, we selected two proteins (SAA4 and ASTL) for validation. We subjected protein from a total of 68 cyst fluids and 68 serum samples to immunoblot. Semi-quantitative protein levels were compared between benign and malignant samples for each fluid compartment.

### SAA4 is significantly increased in ovarian cyst fluids but not in plasma

**Figure 3:**
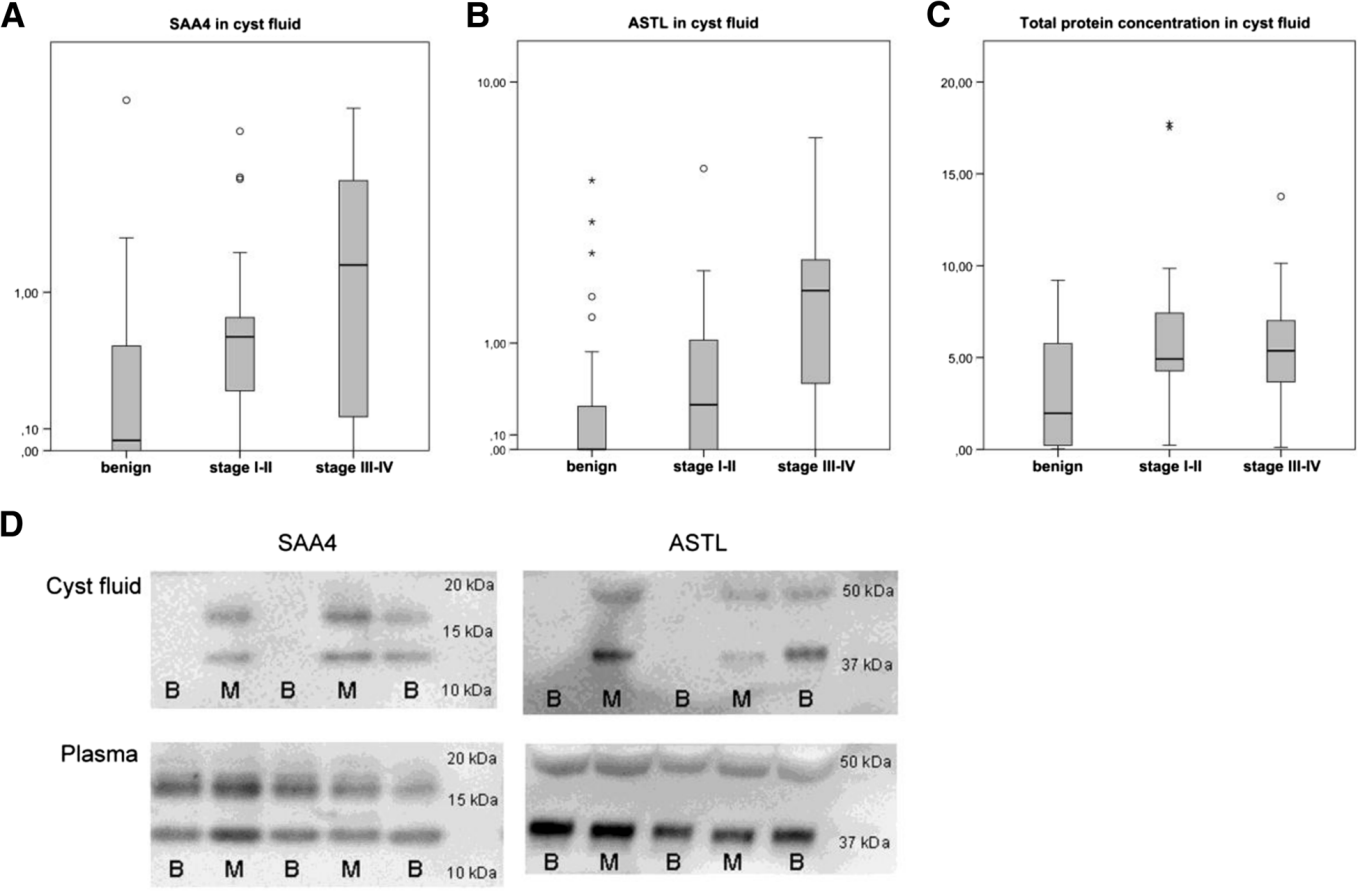
**Immunoblot validation of SAA4 and ASTL in 68 cyst fluid samples with mixed histology.** Protein concentration in cyst fluid in benign, stage I-II, and stage III-IV for **A) SAA4**, malignant samples displayed a significantly higher expression than benign (p=0.001). **B) ASTL,** significantly increased expression in malignant samples (p=0.003) were in contrast to the iTRAQ results. **C) Total protein,** significantly higher in the malignant cohort compared to the benign (p=0.02). **D) Immunoblot expression SAA4 and ASTL** in cyst fluid and plasma. In plasma there was no significant difference between benign and malignant samples for either SAA4 nor ASTL.

The SAA4 (15 kDa) antibody detected two bands, at 13 kDa and 17 kDa (Figure 3D), which were expected according to the manufacturer’s description. The intensity of the two bands correlated well in all samples, and the 13 kDa band was subjected to densitometric scanning. The cyst fluid from patients with malignant disease displayed a significantly higher expression of SAA4 (p = 0.001) compared to the benign samples (Figure 3A), confirming the results from the iTRAQ analysis where the SAA4 expression levels also differed significantly (p = 0.001). The trend of increased expression in higher stages detected in the iTRAQ analysis was persistent in this larger heterogenic sample set (Figures 2A and 3A). SAA4 levels were then examined according to histologic subtype (data not shown). SAA4 were still significantly increased in serous EOC. SAA4 levels were low in simple cysts, mucinous adenoma and all six mucinous carcinomas. SAA4 levels were equally high in both endometrioma and endometrioid EOC. However this is a rather small set of subgroup samples and our results need further validation.

To evaluate the potential of SAA4 as a serological biomarker for ovarian cancer, the protein expression of SAA4 was compared in plasma samples from 68 patients identical with the cohort previously used in the validation of cyst fluid samples. There were, however, no significant differences in expression levels between the benign and malignant plasma samples (p = 0.81; Figure 3D).

### Total protein concentration

The total protein concentration in the cyst fluids was measured and as expected was lower in the benign cohort (median 1.98 mg/ml, range 0.03-9.20) than in the malignant (median 5.26 mg/ml, range 0.12-17.73) (p = 0.02; Figure 3C). To be able to determine whether the differences in SAA4 actually is a reflection of higher protein concentrations we used the samples with more equal protein concentrations from both groups and performed statistical calculations on this more homogenous set of samples. In this cohort, the median concentration of benign samples was 5.25 mg/ml (range 1.33-9.20) (n = 18), and in malignant samples 5.41 mg/ml (range 1.75-17.73) (n = 31). Statistical verification of SAA4 in this cohort revealed that it was still significant (p = 0.013).

### Significant but contradictory results in the ASTL verification and validation

Unexpectedly, the cyst fluid levels of ASTL were significantly higher in malignant cyst fluids (p = 0.003). This was in contrast with our results from the iTRAQ MS analysis were the results showed significant (p < 0.001) lower levels in the malignant samples (Figures 2B and 3B). The ASTL antibody detected two bands at 40 and 48 kDa. The predicted size was expected to be 46 kDa. ASTL levels were then examined according to histologic subtype (data not shown). ASTL levels were increased in serous EOC and endometrioid EOC, but not in clear cell or mucinous EOC. ASTL levels were low in simple cysts, benign serous and mucinous tumor cysts. These results are however difficult to interpret since the two methods did not correlate.

Even though ASTL results from iTRAQ and immunoblot were contradictory we chose to evaluate its potential as a biomarker in blood. There were, however, no significant differences in expression levels between the benign and malignant plasma samples (Figure 3D).

### Verification of the iTRAQ metod

Among the 68 cyst fluids used in the external validation set, two benign serous adenomas and five serous adenocarcinomas of different stages were identical with the iTRAQ sample set and demonstrated good correlation for the SAA4 expression (p = 0.008; data not shown). However, ASTL expression did not correlate within the two methods, which is in line with the significant but contradictory findings (p = 0.58; data not shown).

## Discussion

This study established that there are significant differences in the expression levels of a number of proteins in ovarian cyst fluid when benign and malignant tumors are compared indicating that it might be possible to use this fluid to identify novel biomarkers for ovarian tumor diagnosis. In this study we used a quantitative proteomic technique to analyze sets of fifteen immunodepleted cyst fluids from patients with ovarian serous adenomas and serous adenocarcinomas of different stages. The samples were not pooled in order to see individual differences. Epithelial ovarian cancer consists of at least five different histological subtypes, and no known biomarker covers all histologies as a single marker [24]. To increase our chances of finding a true novel biomarker, we chose only patients with serous histology for the initial proteomic screening. In the verification and validation part of this study, 50% of the included tumors were of serous origin, which is slightly lower than the normal incidence. We used iTRAQ MS, which has a low variance between runs and can take up to seven samples together with a reference sample under identical conditions.

Potential tumor-specific biomarkers are most likely those produced by epithelial ovarian tumor cells or surrounding stroma and secreted into the cyst fluid compartment, and thereafter to lymph vessels and the bloodstream where we can easily detect them. We hypothesize that changes in protein levels can more easily be found in the ovarian cyst fluid in the initial phase of the disease than in serum. And indeed, we could identify 837 different proteins in the cyst fluids after immunodepletion, and 32 of these were significantly differentially expressed between the benign and malignant groups. Fifteen proteins were identified in all five iTRAQ sets; eight were identified in four sets and five were identified in three sets. Several of the proteins identified in this study have previously been identified as potential ovarian cancer biomarkers in both serum and tissue biopsies [20-22], demonstrating that iTRAQ MS of ovarian cyst fluids can be used for the identification of differentially expressed biomarkers for later validation in serum. Interestingly, among these proteins some were expressed with even higher levels among the stage IA tumors compared to stage IIIC tumors (Figure 1).

SAA4, an acute-phase protein, was significantly differentially expressed between the two groups (p = 0.04) in the present study and has previously been suggested to be involved in carcinogenesis [25-27]. This difference between benign and malignant tumors is supported by another study performed by our group, where we analysed benign and malignant samples using SELDI-TOF MS [2]. We had therefore several reasons why SAA4 is an interesting choice for further evaluation as a potential biomarker in ovarian cancer. A group of 68 cyst fluids of heterogeneous histology were subsequently analysed by immunoblot, and the divergence remained between the groups (p = 0.001), which suggests SAA4 as a potential novel biomarker. However SAA4 were negative in most of the mucinous tumors. Interestingly, the increasing levels of SAA4 in relation to tumor progression (stage I – stage III) were detected both in the present study and in our SELDI-TOF MS investigation. The increasing amount of SAA4 in higher stages could suggest that tumors produce acute phase proteins as a response to injury or inflammation itself. Increasing expression levels of SAA1 and SAA4 mRNA and protein have been found from benign to primary and metastatic adenocarcinomas in ovarian tissue sections [27]. The levels of SAA4 in our study also correlated well with the seven samples that were similar within the two methods, iTRAQ and immunoblot. Speculatively, it would be interesting to explore the potential of using SAA4 for imaging diagnostics. We wonder if it would be possible to label an antibody for SAA4 with a nuclide and then screen the patient with PET or some other equipment and be able to verify the presence of a malignant tumor as opposed to a benign cyst.

Astacin-like metalloendopeptidase (ASTL) with a fold change of 8.48 was identified as one of the most interesting proteins from the iTRAQ analysis. ASTL had the largest fold change and it has previously been associated with expression in the ovary and ovarian carcinomas [28]. Even though these results could be questioned early because of only 1–3 peptide recognitions in three of five sets, we aimed to further evaluate ASTL. The external validation of 68 cyst fluids revealed a significant but reverse relationship between the expression of ASTL and malignancy compared to iTRAQ data. These indistinguishable results made us question whether it really was ASTL that we detected in the iTRAQ MS analysis, since the detected peptide sequence was identical in all sample hits for ASTL.

GRP78 has previously been associated with ovarian cancer [29,30], and our results from the iTRAQ analysis indicate that this protein may potentially be a good biomarker for ovarian tumors since there was no overlap in expression levels between the benign and the malignant samples (p = 0.005). This finding is well in line with GRP78 being located in the endoplasmic reticulum in normal cells and on the surface of cancerous cells, making it interesting as a target for cancer diagnostics and therapies [31]. Taxol coupled to GRP78 antibody has been shown to suppress tumor cell growth in vitro [32]. Unfortunately, we could not detect GRP78 with the commercial antibody we tried (data not shown).

A number of other interesting proteins were identified by our proteomic screening and are suitable for further investigation. In this study we compared the benign tumors with all malignant. The next step should perhaps be to evaluate the proteins that increase in early stage EOC i.e. Peroxiredoxinsare H_2_O_2_ scavenging antioxidant (PRDX), Clathrin heavy chain 1 (CLTC), or complement component 4 binding protein alpha (C4BPA). The “depleted” albumin displayed significantly differentially expressed levels albumin between the benign and malignant samples with higher levels in the benign samples. Studies have suggested that albumin is a potential biomarker for survival of cancer. For example, Parker et al. as well as McMillan et al. described that patients diagnosed with epithelial ovarian cancer who have higher levels of albumin have a better chance of survival [33,34]. In our study, S100A8 and S100A9 were identified, and levels of these proteins were higher among the malignant samples. Both S100 proteins have previously been described in several tumor types and suggested to be involved in ovarian and colorectal cancer [35-37]. S100A8 and S100A9, also known as Calgranulins A and B, were first identified in cyst fluid and serum as up regulated in ovarian cancer, but absent or negative in benign cysts [38]. Up regulation in ovarian tissue and peritoneal fluid has been reported [35]. S100A8 and S100A9 are involved in numerous inflammation and carcinogenesis cellular processes and can be used as cancer biomarkers, but are not specific markers for ovarian cancer [39]. Furthermore we identified SPARCL1 with higher expression levels among the benign samples than the malignant ones (p = 0.01), and previous investigations discussed this protein’s possible involvement as a suppressor of a variety of tumor types [40,41]. To improve the diagnosis of EOC we need panels of biomarkers that take into account the great heterogeneity of the disease, with variations in molecular and biological behavior as well as different histology. Our work will continue to study a number of interesting proteins that we identified as differentially expressed between early cancer and benign tumors.

## Conclusions

Fluid from ovarian cysts connected directly to the primary tumor that harbors many possible new tumor-specific biomarkers. With iTRAQ MS on cyst fluid from serous ovarian tumors, we identified 32 differentially expressed proteins comparing benign and malignant cysts. Some of these proteins have recently been suggested as novel biomarkers for ovarian cancer [42-45], and additionally quite a few have previously been described as cancer related. Among a number of interesting proteins differently expressed, two candidate markers were validated to verify the iTRAQ method.

## Materials and methods

### Collection of the material

Cyst fluids and blood were collected prospectively and consecutively from patients diagnosed from March 2001 to September 2010 with suspicious cystic pelvic tumors Patients were included when they were admitted for an operation to the section for gynecologic oncology surgery of Sahlgrenska University hospital, Gothenburg, Sweden. According to our protocol, blood samples were taken after anesthesia but before surgery, and cyst fluids were collected after removal of the cysts from the abdomen. All samples were put directly in 4°C for 15–30 minutes, centrifuged, aliquoted into eppendorf tubes, and stored in −80°C within 30–60 minutes after collection. Samples used in this study had experienced one freeze-thaw cycle. Removed tumors were examined by an experienced pathologist for histology and grade and staged (I-IV) according to FIGO standards. The local ethical committee at the University of Gothenburg approved the study, and each patient gave her informed, written consent.

### Sample selection

To obtain a homogenous group of samples in the iTRAQ MS analysis, only serous ovarian adenomas and adenocarcinomas, the most common form of epithelial ovarian cancers, were included from our ovarian cyst fluid biobank. A total of 15 cyst fluid samples were analyzed with iTRAQ (Table 1). The following verification and validation included cyst fluid and plasma samples (n = 136) from 68 patients, with mix of all common ovarian histologies. We included 32 patients with benign cysts and 36 patients with EOC (Table 3). Seven samples from the iTRAQ analysis were included in the verification set, two benign and five malignant samples. In the validation step, the cohort consisted of tumors of different histologies, and serous carcinoma represented 50% of the malignant samples.

### Sample preparation for MS analysis

Mass spectrometry analysis was performed at the Proteomic Core Facility at the University of Gothenburg. Our previous data showed that proteins, which are abundant in the blood, are even more abundant in cyst fluid [18,2]. Thus, beforehand removal of these proteins from the cyst fluid is required for the MS analysis to be able to detect potential tumor-specific biomarkers. In this study, we used a depletion method before MS and labeling by isobaric tag for relative and absolute quantitation (iTRAQ).

All 15 samples (50 μl each) were filtered using a 0.22 μm spin filter at 2000 rpm. The protein content was determined by Pierce BCA Protein Assay (Thermo Fisher Scientific, Rockford, IL, USA). Depletion of human albumin and IgG were performed (25 μl of each sample) using the Qproteome Albumin/IgG Depletion Kit (Qiagen, Valencia, CA, USA). The protein concentration was determined once more by Pierce BCA Protein Assay (Thermo Fisher Scientific).

100 μg of each sample was withdrawn and diluted to 200 μl. Non-protein impurities were removed by quantitative precipitation clean-up using ProteoExtract® Protein Precipitation (Calbiochem, San Diego, CA, USA). The pellets were dissolved in iTRAQ® Dissolution Buffer with the addition of 1 μl 2% SDS (iTRAQ®, Applied Biosystems, Foster City, CA, USA), and the samples were digested with trypsin (Promega, Madison, WI, USA), reduced, and alkylated. All the 15 samples included in the analysis were pooled together and used as a standard for the iTRAQ analysis in each run. Each four-plex set consisted of one pooled standard sample and three different patient samples labeled with the iTRAQ® reagent 114, 115, 116, and 117 respectively, following the manufacturer’s instructions (Applied Biosystems).

### Strong cation exchange chromatography (SCX) of iTRAQ-labeled peptides

The concentrated peptides were acidified by 10% formic acid and diluted with SCX solvent A (25 mM ammonium formate, pH 2.8, 20% acetonitrile [ACN]) and injected onto a PolySULFOETHYL A SCX column (2.1 mm i.d. × 10 cm length, 5 μm particle size, 300 Å pore size). SCX chromatography and fractionation was carried out on an ÄKTA purifier system (GE Healthcare, Buckinghamshire, UK) at 0.25 mL/min flow rate using the following gradient: 0% B (500 mM ammonium formate, pH 2.8, 20% ACN) for 5 min; 0-40% B for 20 min; 40-100% B for 10 min; and 100% B held for 10 min. UV absorbance at 254 and 280 nm was monitored while fractions were collected at 0.5 mL intervals and dried down in a SpeedVac. The peptide-containing fractions (10) were desalted on PepClean C18 spin columns according to the manufacturer’s instructions (Thermo Fisher Scientific).

### LC-MS/MS analysis on LTQ-Orbitrap

The desalted and dried fractions were reconstituted into 0.1% formic acid and analyzed on a LTQ-Orbitrap XL (Thermo Fisher Scientific) interfaced with an in-house-constructed nano-LC system, described elsewhere [46]. Briefly, two-microliter sample injections were made with an HTC-PAL autosampler (CTC Analytics AG, Zwingen, Switzerland) connected to an Agilent 1200 binary pump (Agilent Technologies, Palo Alto, CA, USA). The peptides were trapped on a precolumn (45 × 0.075 mm i.d.) and separated on a reversed phase column, 200 × 0.050 mm. Both columns are packed in-house with 3 μm Reprosil-Pur C_18_-AQ particles. The flow through from the analytical column was reduced by a split to approximately 100 nl/min, and the gradient was as follows: 0–5 min 0.1% formic acid; 6–103 min 7-32% ACN 0.1% formic acid; and 103–105 min 80% ACN 0.1% formic acid.

LTQ-Orbitrap settings were as follows: spray voltage 1.4 kV, 1 microscan for MS1 scans at 60 000 resolution (m/z 400), full MS mass range m/z 400–2000. The LTQ-Orbitrap was operated in a data-dependent mode, that is, one MS1 FTMS scan precursor ions followed by CID (collision induced dissociation) and HCD (high energy collision dissociation) MS2 scans of the three most abundant doubly or triply protonated ions in each FTMS scan. The settings for the MS2 were as follows: 1 microscan for HCD-MS2 at 7500 resolution (at m/z 400), mass range m/z 100–2000 with a collision energy of 50%; 1 microscan for CID-MS2 with a collision energy of 30%.

### Database search and iTRAQ quantification

MS raw data files from all ten SCX fractions for one four-plex iTRAQ set were merged for relative quantification and identification using Proteome Discoverer version 1.1 (Thermo Fisher Scientific). A database search for each of the five sets was performed by Mascot search engine using the following criteria: homo sapiens in Swissprot version 57.15, MS peptide tolerance as 5 ppm, MS/MS tolerance as 0.05 Da, trypsin digestion allowing 2 missed cleavages with variable modifications; methionine oxidation, cysteine methylthiolation, tyrosine iTRAQ4plex (+144 Da) and fixed modifications; and N-terminal iTRAQ4plex, lysine iTRAQ4plex. The detected protein threshold in the software was set to 95% confidence, and identified proteins were grouped by those sharing the same sequences to minimize redundancy.

For iTRAQ quantification, the ratios of iTRAQ reporter ion intensities in MS/MS spectra (m/z 114.11-117.11) from the raw data sets were used to calculate fold changes (FC) between samples. Ratios were derived by Proteome Discoverer version 1.1 using the following criteria: fragment ion tolerance as 50 ppm for the most confident centroid peak; iTRAQ reagent purity corrections factors are used and missing values are replaced with minimum intensity. Only peptides unique to a given protein were considered for relative quantitation, excluding those common to other isoforms or proteins of the same family. The ratios were normalized to the mean value of the 50 ratios identified with highest number of peptides.

### Immunoblotting

The protein concentrations of 68 cyst fluid and 68 plasma samples were determined with the Micro BCA protein assay kit according to the manufacturer’s instructions (Thermo Fisher Scientific). The cyst fluid samples were diluted in H_2_O 1:10 and plasma samples 1:5, and 2.5 μl of each sample was diluted in (SDS) sample buffer with a reducing agent (Invitrogen). After heating at 70°C for 10 minutes, the samples were loaded on SDS-PAGE (NuPAGE 4–12% Bis-Tris Gel, Invitrogen Ltd., Paisley, UK) and separated by electrophoresis using MES SDS running buffer (Invitrogen). Proteins were transferred to polyvinyl difluoride membranes using the iBlot dry blotting system (Invitrogen). Membranes were blocked in 5% non-fat milk in 10 mM phosphate buffered saline (PBS) containing 0,05% Tween 20. The membranes were incubated overnight at 4°C with PBS containing 0,05% Tween 20 and the following primary antibodies: serum amyloid A-4 protein (SAA4) purified MaxPab mouse polyclonal antibody (1:1000, Abnova, Taiwan); astacin-like metalloendopeptidase (ASTL) (N-12) goat polyclonal (1:800, Santa Cruz Biotechnology, Inc., Santa Cruz, CA, USA); and 78 kDa glucose-regulated protein (GRP78) (N-20) goat polyclonal (1:500, Santa Cruz Biotechnology). Precision plus protein WesternC standards (Bio-Rad, Hercules, CA, USA) were used as molecular weight markers. Immunoreactivity protein was visualized by chemiluminescence using peroxidase-labeled secondary antimouse (1:10 000, GE Healthcare), secondary antigoat (1:15 000, Santa Cruz Biotechnology) detected with chemiluminescent ECL Advance (GE Healthcare). Immunoblotted membranes were exposed using a LAS-1000 (Fujifilm, Minato-ku Tokyo, Japan). Individual bands were quantified from the membrane images by densitometry using the Quantity One software program (Bio-Rad). An internal reference sample, the same on each blot, was used as a standard for quantification of bands detected in cyst fluid samples and was given the value 1 [47].

### Statistical analysis

The normalized iTRAQ MS peak ratios were transformed to Log_2_ values. Protein entries with only a single peptide hit and proteins only detected in one or two sets, as well as various entries corresponding to IgG isoforms, were not included in the analysis. Differences between benign and malignant samples were compared using t-test and a list of significant results presenting proteins with p < 0.05 and at least a 1.8 fold change were generated.

For the validation assay, the statistical differences in protein expressions were calculated using the Mann–Whitney U test, and the relation between expressions measured with iTRAQ MS. Immunoblotting was evaluated with bivariate correlation using Spearman correlation coefficient. A value of p < 0.05 was considered to be significant.

## Abbreviations

ASTL: Astacin-like metalloendopeptidase; S100A8/A9: S100 calcium binding proteins A8/A or calgranulins A and B; EOC: Epithelial ovarian cancer; FIGO: International Federation of Gynecology and Obstetrics; GRP78: 78 kDa glucose-regulated protein; HE4: Human epididymis protein 4; iTRAQ: Isobaric tags for relative and absolute quantification; LC: Liquid chromatography; LTQ: Linear trap quadrupole; MS: Mass spectrometry; SAA4: Serum amyloid A4; SCX: Strong cation exchange chromatography; SPARCL1: Secreted protein, acidic and rich in cysteine-like 1; TMT: Tandem mass tag.

## Competing interests

The authors declare that they have no competing interests.

## Authors’ contributions

BK has been involved in planning of study, collection of material, evaluating data and manuscript writing. KL has been involved in evaluating and analyzing the data and writing the manuscript. KP has been running the immunoblot and evaluating data as well as involvement in the writing process. EC has been involved in the planning of the study, iTRAQ performance and data evaluation. KS has been responsible for the project and been involved in the planning of the study, the evaluation of the data as well as being involved in writing the manuscript. All authors read and approved the final manuscript.

## References

[B1] Socialstyrelsen CfEoTSNBoHaW Causes of death 2008 2010 The Swedish National Board of Health and Welfare www.socialstyrelsen.se/statistik/statistikefteramne/cancer

[B2] Kristjansdottir B Partheen K Fung ET Marcickiewicz J Yip C Brannstrom M Sundfeldt K Ovarian cyst fluid is a rich proteome resource for detection of new tumor biomarkers Clinical Proteomics 2012 10 1 14 10.1186/1559-0275-9-14 23268721 PMC3552982

[B3] Heintz AP Odicino F Maisonneuve P Quinn MA Benedet JL Creasman WT Ngan HY Pecorelli S Beller U Carcinoma of the ovary. FIGO 6th annual report on the results of treatment in gynecological cancer Int J Gynaecol Obstet 2006 10 Suppl 1 S161 S192 17161157 10.1016/S0020-7292(06)60033-7

[B4] van Nagell JR Jr DePriest PD Ueland FR DeSimone CP Cooper AL McDonald JM Pavlik EJ Kryscio RJ Ovarian cancer screening with annual transvaginal sonography: findings of 25,000 women screened Cancer 2007 10 9 1887 1896 10.1002/cncr.22594 17373668

[B5] MacDonald ND Jacobs IJ Is there a place for screening in ovarian cancer? Eur J Obstet Gynecol Reprod Biol 1999 10 2 155 157 10.1016/S0301-2115(98)00219-X 10206408

[B6] Shih Ie M Kurman RJ Molecular pathogenesis of ovarian borderline tumors: new insights and old challenges Clin Cancer Res: An Official J Am Assoc Cancer Res 2005 10 20 7273 7279 10.1158/1078-0432.CCR-05-0755 16243797

[B7] Markman M The role of CA-125 in the management of ovarian cancer Oncologist 1997 10 1 6 9 10388024

[B8] Markman M Federico M Liu PY Hannigan E Alberts D Significance of early changes in the serum CA-125 antigen level on overall survival in advanced ovarian cancer Gynecol Oncol 2006 10 1 195 198 10.1016/j.ygyno.2006.02.024 16595148

[B9] Meyer T Rustin GJ Role of tumour markers in monitoring epithelial ovarian cancer Br J Cancer 2000 10 9 1535 1538 10789720 10.1054/bjoc.2000.1174 PMC2363391

[B10] Kolwijck E Span PN Thomas CM Bulten J Sweep FC Massuger LF Prognostic value of CA 125 in ovarian cyst fluid of patients with epithelial ovarian cancer Oncol Rep 2010 10 2 579 584 20043124

[B11] Kolwijck E Zusterzeel PL Roelofs HM Hendriks JC Peters WH Massuger LF GSTP1-1 in ovarian cyst fluid and disease outcome of patients with ovarian cancer Cancer Epidemiology, Biomarkers & Prevention: A Publication of the American Association for Cancer Research, Cosponsored by the American Society of Preventive Oncology 2009 10 8 2176 2181 10.1158/1055-9965.EPI-09-0098 19661073

[B12] Woolas RP Xu FJ Jacobs IJ Yu YH Daly L Berchuck A Soper JT Clarke-Pearson DL Oram DH Bast RC Elevation of multiple serum markers in patients with stage I ovarian cancer J Natl Cancer Inst 1993 10 21 1748 1751 10.1093/jnci/85.21.1748 8411259

[B13] Nolen B Velikokhatnaya L Marrangoni A De Geest K Lomakin A Bast RC Jr Lokshin A Serum biomarker panels for the discrimination of benign from malignant cases in patients with an adnexal mass Gynecol Oncol 2010 10 3 440 445 10.1016/j.ygyno.2010.02.005 20334903 PMC2873171

[B14] Moore RG Brown AK Miller MC Skates S Allard WJ Verch T Steinhoff M Messerlian G DiSilvestro P Granai CO The use of multiple novel tumor biomarkers for the detection of ovarian carcinoma in patients with a pelvic mass Gynecol Oncol 2008 10 2 402 408 10.1016/j.ygyno.2007.10.017 18061248

[B15] Hellstrom I Raycraft J Hayden-Ledbetter M Ledbetter JA Schummer M McIntosh M Drescher C Urban N Hellstrom KE The HE4 (WFDC2) protein is a biomarker for ovarian carcinoma Cancer Res 2003 10 13 3695 3700 12839961

[B16] Havrilesky LJ Whitehead CM Rubatt JM Cheek RL Groelke J He Q Malinowski DP Fischer TJ Berchuck A Evaluation of biomarker panels for early stage ovarian cancer detection and monitoring for disease recurrence Gynecol Oncol 2008 10 3 374 382 10.1016/j.ygyno.2008.04.041 18584856

[B17] Petri AL Simonsen AH Yip TT Hogdall E Fung ET Lundvall L Hogdall C Three new potential ovarian cancer biomarkers detected in human urine with equalizer bead technology Acta Obstet Gynecol Scand 2009 10 1 18 26 10.1080/00016340802443830 19023702

[B18] Ivarsson K Runesson E Sundfeldt K Haeger M Hedin L Janson PO Brannstrom M The chemotactic cytokine interleukin-8–a cyst fluid marker for malignant epithelial ovarian cancer? Gynecol Oncol 1998 10 3 420 423 10.1006/gyno.1998.5198 9887242

[B19] Sundfeldt K Ivarsson K Rask K Haeger M Hedin L Brannstrom M Higher levels of soluble E-cadherin in cyst fluid from malignant ovarian tumours than in benign cysts Anticancer Res 2001 10 1A 65 70 11299791

[B20] Boylan KL Andersen JD Anderson LB Higgins L Skubitz AP Quantitative proteomic analysis by iTRAQ(R) for the identification of candidate biomarkers in ovarian cancer serum Proteome Sci 2010 10 31 10.1186/1477-5956-8-31 20546617 PMC2893134

[B21] Gagne JP Ethier C Gagne P Mercier G Bonicalzi ME Mes-Masson AM Droit A Winstall E Isabelle M Poirier GG Comparative proteome analysis of human epithelial ovarian cancer Proteome science 2007 10 16 10.1186/1477-5956-5-16 17892554 PMC2072939

[B22] Waldemarson S Krogh M Alaiya A Kirik U Schedvins K Auer G Hansson KM Ossola R Aebersold R Lee H Protein expression changes in ovarian cancer during the transition from benign to malignant J Proteome Res 2012 10 5 2876 2889 10.1021/pr201258q 22471520

[B23] Ryu JK Woo SM Hwang JH Jeong JB Yoon YB Park IA Han JK Kim YT Cyst fluid analysis for the differential diagnosis of pancreatic cysts Diagn Cytopathol 2004 10 2 100 105 10.1002/dc.20085 15282721

[B24] Toyama A Suzuki A Shimada T Aoki C Aoki Y Umino Y Nakamura Y Aoki D Sato TA Proteomic characterization of ovarian cancers identifying annexin-A4, phosphoserine aminotransferase, cellular retinoic acid-binding protein 2, and serpin B5 as histology-specific biomarkers Cancer Sci 2012 10 4 747 755 10.1111/j.1349-7006.2012.02224.x 22321069 PMC7659196

[B25] Gutfeld O Prus D Ackerman Z Dishon S Linke RP Levin M Urieli-Shoval S Expression of serum amyloid A, in normal, dysplastic, and neoplastic human colonic mucosa: implication for a role in colonic tumorigenesis The J histochemistry and Cytochemistry: Official J Histochemistry Soc 2006 10 1 63 73 10.1369/jhc.5A6645.2005 16116035

[B26] Michaeli A Finci-Yeheskel Z Dishon S Linke RP Levin M Urieli-Shoval S Serum amyloid A enhances plasminogen activation: implication for a role in colon cancer Biochem Biophys Res Commun 2008 10 2 368 373 10.1016/j.bbrc.2008.01.079 18237545

[B27] Urieli-Shoval S Finci-Yeheskel Z Dishon S Galinsky D Linke RP Ariel I Levin M Ben-Shachar I Prus D Expression of serum amyloid a in human ovarian epithelial tumors: implication for a role in ovarian tumorigenesis The J Histochemistry and Cytochemistry: Official J Histochemistry Soc 2010 10 11 1015 1023 10.1369/jhc.2010.956821 PMC2958134 20713982

[B28] Quesada V Sanchez LM Alvarez J Lopez-Otin C Identification and characterization of human and mouse ovastacin: a novel metalloproteinase similar to hatching enzymes from arthropods, birds, amphibians, and fish J Biol Chem 2004 10 25 26627 26634 10.1074/jbc.M401588200 15087446

[B29] Taylor DD Gercel-Taylor C Parker LP Patient-derived tumor-reactive antibodies as diagnostic markers for ovarian cancer Gynecol Oncol 2009 10 1 112 120 10.1016/j.ygyno.2009.06.031 19647308 PMC2760307

[B30] Cohen M Petignat P Purified autoantibodies against glucose-regulated protein 78 (GRP78) promote apoptosis and decrease invasiveness of ovarian cancer cells Cancer Lett 2011 10 1 104 109 10.1016/j.canlet.2011.05.022 21658840

[B31] Jakobsen CG Rasmussen N Laenkholm AV Ditzel HJ Phage display derived human monoclonal antibodies isolated by binding to the surface of live primary breast cancer cells recognize GRP78 Cancer Res 2007 10 19 9507 9517 10.1158/0008-5472.CAN-06-4686 17909061

[B32] Kim Y Lillo AM Steiniger SC Liu Y Ballatore C Anichini A Mortarini R Kaufmann GF Zhou B Felding-Habermann B Targeting heat shock proteins on cancer cells: selection, characterization, and cell-penetrating properties of a peptidic GRP78 ligand Biochemistry 2006 10 31 9434 9444 10.1021/bi060264j 16878978

[B33] Parker D Bradley C Bogle SM Lay J Masood M Hancock AK Naylor B Price JJ Serum albumin and CA125 are powerful predictors of survival in epithelial ovarian cancer Br J Obstet Gynaecol 1994 10 10 888 893 10.1111/j.1471-0528.1994.tb13550.x 7999691

[B34] McMillan DC Elahi MM Sattar N Angerson WJ Johnstone J McArdle CS Measurement of the systemic inflammatory response predicts cancer-specific and non-cancer survival in patients with cancer Nutr Cancer 2001 10 1–2 64 69 12094630 10.1080/01635581.2001.9680613

[B35] Cortesi L Rossi E Della Casa L Barchetti A Nicoli A Piana S Abrate M La Sala GB Federico M Iannone A Protein expression patterns associated with advanced stage ovarian cancer Electrophoresis 2011 10 15 1992 2003 10.1002/elps.201000654 21728179

[B36] Ju W Yoo BC Kim IJ Kim JW Kim SC Lee HP Identification of genes with differential expression in chemoresistant epithelial ovarian cancer using high-density oligonucleotide microarrays Oncol Res 2009 10 2–3 47 56 20066894 10.3727/096504009789954672

[B37] Kim HJ Kang HJ Lee H Lee ST Yu MH Kim H Lee C Identification of S100A8 and S100A9 as serological markers for colorectal cancer J Proteome Res 2009 10 3 1368 1379 10.1021/pr8007573 19186948

[B38] Ott HW Lindner H Sarg B Mueller-Holzner E Abendstein B Bergant A Fessler S Schwaerzler P Zeimet A Marth C Calgranulins in cystic fluid and serum from patients with ovarian carcinomas Cancer Res 2003 10 21 7507 7514 14612552

[B39] Kostakis ID Cholidou KG Kallianidis K Perrea D Antsaklis A The role of calprotectin in obstetrics and gynecology Eur J Obstet Gynecol Reprod Biol 2010 10 1 3 9 10.1016/j.ejogrb.2010.03.006 20378239

[B40] Esposito I Kayed H Keleg S Giese T Sage EH Schirmacher P Friess H Kleeff J Tumor-suppressor function of SPARC-like protein 1/Hevin in pancreatic cancer Neoplasia 2007 10 1 8 17 10.1593/neo.06646 17325739 PMC1803032

[B41] Yu SJ Yu JK Ge WT Hu HG Yuan Y Zheng S SPARCL1, Shp2, MSH2, E-cadherin, p53, ADCY-2 and MAPK are prognosis-related in colorectal cancer World J Gastroenterol 2011 10 15 2028 2036 10.3748/wjg.v17.i15.2028 21528083 PMC3082758

[B42] Hogdall C Fung ET Christensen IJ Nedergaard L Engelholm SA Petri AL Risum S Lundvall L Yip C Pedersen AT A novel proteomic biomarker panel as a diagnostic tool for patients with ovarian cancer Gynecol Oncol 2011 10 2 308 313 10.1016/j.ygyno.2011.07.018 21855971

[B43] Maines-Bandiera S Woo MM Borugian M Molday LL Hii T Gilks B Leung PC Molday RS Auersperg N Oviductal glycoprotein (OVGP1, MUC9): a differentiation-based mucin present in serum of women with ovarian cancer Int J Gynecol Cancer 2010 10 1 16 22 10.1111/IGC.0b013e3181bcc96d 20130498

[B44] Biade S Marinucci M Schick J Roberts D Workman G Sage EH O’Dwyer PJ Livolsi VA Johnson SW Gene expression profiling of human ovarian tumours Br J Cancer 2006 10 8 1092 1100 10.1038/sj.bjc.6603346 16969345 PMC2360705

[B45] Abdel-Azeez HA Labib HA Sharaf SM Refai AN HE4 and mesothelin: novel biomarkers of ovarian carcinoma in patients with pelvic masses Asian Pac J Cancer Prev 2010 10 1 111 116 20593939

[B46] Carlsohn E Nystrom J Karlsson H Svennerholm AM Nilsson CL Characterization of the outer membrane protein profile from disease-related Helicobacter pylori isolates by subcellular fractionation and nano-LC FT-ICR MS analysis J Proteome Res 2006 10 11 3197 3204 10.1021/pr060181p 17081072

[B47] Zhu Y Brannstrom M Janson PO Sundfeldt K Differences in expression patterns of the tight junction proteins, claudin 1, 3, 4 and 5, in human ovarian surface epithelium as compared to epithelia in inclusion cysts and epithelial ovarian tumours Int J Cancer J Int Du Cancer 2006 10 8 1884 1891 10.1002/ijc.21506 16287068

